# Adverse Pregnancy Outcomes and Cardiovascular Health Among Offspring in Early Adulthood

**DOI:** 10.1001/jamanetworkopen.2026.6783

**Published:** 2026-05-14

**Authors:** Emily L. Lam, Abigail M. Gauen, Sadiya S. Khan, Alexa Freedman, James H. Stein, Kartik K. Venkatesh, Elisabeth Tawa, Hacer Savas, Noreen Goldman, Daniel A. Notterman, Donald M. Lloyd-Jones, Norrina B. Allen, Nilay S. Shah

**Affiliations:** 1 Department of Medicine, Northwestern University Feinberg School of Medicine, Chicago, Illinois; 2 Department of Preventive Medicine, Northwestern University Feinberg School of Medicine, Chicago, Illinois; 3 Department of Medicine, University of Wisconsin School of Medicine and Public Health, Madison; 4 Department of Obstetrics and Gynecology, The Ohio State University College of Medicine, Columbus; 5 Department of Molecular Biology, Princeton University, Princeton, New Jersey; 6 Office of Population Research, Princeton University, Princeton, New Jersey

## Abstract

**Importance:**

Adverse pregnancy outcomes (APOs) confer risk for maternal cardiovascular disease, but the role of APOs in cardiovascular health (CVH) of offspring across the life course is unknown.

**Objective:**

To examine associations of APO exposure with CVH and early arterial injury among offspring in young adulthood.

**Design, Setting, and Participants:**

This longitudinal cohort study used data from the Future of Families and Child Well-Being Study and Future of Families—Cardiovascular Health Among Young Adults study, conducted in 20 US cities from February 1998 to September 2000, with follow-up until September 2023. Participants were mother-child dyads enrolled at the child’s birth; offspring were followed up until young adulthood (year 22). Data were analyzed from June 2024 to June 2025.

**Exposures:**

Hypertensive disorders of pregnancy (HDP), gestational diabetes (GD), and preterm birth (PTB), ascertained by pregnancy medical records.

**Main Outcomes and Measures:**

Offspring CVH assessed by the American Heart Association’s Life’s Essential 8 (LE8) score, individual clinical cardiovascular risk factors, and arterial injury assessed by carotid ultrasonography at year 22 after birth.

**Results:**

Among 1333 offspring participants (729 [55%] female; mean [SD] age in early adulthood, 22.4 [0.7] years), 128 (10%) had HDP exposure, 67 (5%) had GD exposure, and 137 (10%) had PTB exposure. In analyses adjusted for sociodemographic factors, HDP exposure was associated with higher body mass index (adjusted β, 2.80; 95% CI, 1.07-4.53), higher diastolic blood pressure (adjusted β, 2.29; 95% CI, 0.17-4.41), and higher glycated hemoglobin (HbA_1c_) level (adjusted β, 0.21; 95% CI, 0.02-0.41) in early adulthood. PTB was associated with higher HbA_1c_ level (adjusted β, 0.29; 95% CI, 0.15-0.43). GD exposure was associated with a lower LE8 blood pressure score (adjusted β, −6.59; 95% CI, −13.95 to −0.16). HDP exposure was associated with higher mean carotid intima-media thickness (adjusted β, 0.02; 95% CI, 0.01-0.03) and lower carotid grayscale median (adjusted β, −3.68; 95% CI, −6.30 to −1.05). Exposure to GD was associated with higher mean carotid intima-media thickness (adjusted β, 0.02; 95% CI, 0.00-0.04).

**Conclusions and Relevance:**

In this longitudinal cohort study of mother-child dyads enrolled at the child’s birth, exposure to APOs during gestation, particularly HDP, was associated with suboptimal CVH and arterial injury among offspring in early adulthood. The findings suggest optimizing pregnancy health may support offspring CVH into early adulthood.

## Key Points

**Question:**

Is exposure to hypertensive disorders of pregnancy, gestational diabetes, or preterm birth associated with cardiovascular health among offspring in young adulthood?

**Findings:**

In this cohort study of 1333 mother-child dyads enrolled at the child’s birth, exposure to hypertensive disorders of pregnancy was associated with higher body mass index, diastolic blood pressure, and glycated hemoglobin levels and with early arterial injury among offspring at 22 years.

**Meaning:**

The findings suggest offspring exposure to adverse pregnancy outcomes, particularly hypertensive disorders during gestation, may be associated with worse cardiovascular health and early arterial injury in young adulthood.

## Teaser

This cohort study explores whether adverse pregnancy outcomes, including preterm birth, hypertensive disorders of pregnancy, and gestational diabetes, are associated with worse cardiovascular health outcomes among offspring in young adulthood.

## Introduction

Adverse pregnancy outcomes (APOs), such as hypertensive disorders of pregnancy (HDP), gestational diabetes (GD), and preterm birth (PTB), affect almost 1 in 4 pregnancies in the US.^1,2^ National surveillance indicates that rates of each APO increased annually by 3.6% for HDP between 1989 and 2020,^3^ 3.7% for GD between 2011 and 2019,^4^ and 1.4% for PTB between 2014 and 2022.^5^ Emerging data indicate that APOs are associated with the long-term cardiovascular health (CVH) of the person giving birth, including higher risk for metabolic risk factors,^6,7^ image-identified coronary artery disease,^8^ incident ischemic heart disease events,^9^ acute coronary syndrome events,^10^ and mortality later in life.^11^

However, few studies have investigated the long-term consequences of suboptimal maternal health on offspring CVH. David Barker’s hypothesis, first proposed in the late 1980s, posited that low birth weight is associated with higher risk of ischemic heart disease in adulthood.^12^ His work laid the foundation for the broader Developmental Origins of Health and Disease framework, which emphasizes that exposures during critical periods of early development, particularly in utero, may have lasting effects on long-term health outcomes.^13^ The mechanisms by which maternal APOs are associated with offspring CVH remain an area of active investigation.^14^ In the Hyperglycemia and Adverse Pregnancy Outcome (HAPO) study, HDP, GD, and maternal pregnancy cardiometabolic risk factors were associated with worse CVH in early adolescence among offspring, as measured by a limited set of offspring clinical measures, including body mass index (BMI), blood pressure, total cholesterol level, and glucose level.^7,15^

The overall burden of CVH may be summarized by the American Heart Association’s composite measure, Life’s Essential 8 (LE8), which accounts for an individual’s levels of 8 leading cardiovascular risk factors.^16^ LE8 CVH scores relate to the presence of subclinical atherosclerosis in young adults measured by carotid ultrasonography; worse LE8 scores have been associated with higher carotid intima-media thickness (CIMT), greater arterial stiffness, and lower carotid grayscale median (indicating more lipid deposition).^17^ In this study, we evaluated the association of APO exposure with cardiovascular risk factors, composite CVH as measured by LE8, and early subclinical arterial injury identified by carotid ultrasonography in offspring in early adulthood. Quantifying these associations informs investigation of how adverse CVH may be transmitted across generations and may guide interventions before and during pregnancy as critical time periods to promote offspring health.

## Methods

### Participants and Setting

The Future of Families and Child Well-Being Study (FFCWS), a longitudinal cohort study of mother-child dyads enrolled at the child’s birth, was conducted in 20 large US cities from February 1998 to September 2000, with offspring followed through early adulthood. FFCWS oversampled individuals born to mothers who were not married and included a high proportion of participants from low-income families and who belonged to minoritized racial and ethnic communities. Details of the FFCWS design have been previously described in detail.^18^ The Future of Families—Cardiovascular Health Among Young Adults (FF-CHAYA) study, conducted from September 2021 to September 2023, was an FFCWS ancillary study composed of FFCWS offspring participants who had comprehensive measures of CVH and arterial injury evaluated after FFCWS study year 22 (between 2 months and 2 years after the FFCWS year 22 visit).^19^ The Princeton University institutional review board approved the FFCWS and FF-CHAYA studies and the current study. Written informed consent was obtained from parents in the baseline FFCWS and from participants themselves in the FF-CHAYA follow-up study, which also applied to use of participants’ data from FFCWS. Reporting followed the Strengthening the Reporting of Observational Studies in Epidemiology (STROBE) reporting guideline.

Participants in the FF-CHAYA ancillary study attended an in-person examination and completed self-administered questionnaires querying demographic information (including self-reported race and ethnicity); medical history; and health behaviors including dietary habits, physical activity, and nicotine exposure. Anthropometric measurements, including height and weight, were obtained using standardized protocols with participants wearing lightweight clothing. BMI was calculated as weight in kilograms divided by height in meters squared. Phlebotomy was performed for the measurement of standard lipid panels, nonfasting blood glucose, and glycated hemoglobin (HbA_1c_) level. Blood samples were frozen and shipped to the University of Vermont’s Laboratory for Clinical Biochemistry Research. Blood pressure was measured 3 times, 1 minute apart, using automated oscillometric devices (HEM-907XL [OMRON Healthcare, Inc]) after the participants rested for 5 minutes with the cuff placed on their upper arm, per standardized protocols. The mean of the second and third blood pressure measurements was used. Race and ethnicity were included in the current analysis because rates of adverse pregnancy outcomes vary across racial and ethnic groups in the US; categories were Hispanic, non-Hispanic Black (hereafter, *Black*), non-Hispanic White (hereafter, *White*), and other (included multiracial and any other self-reported categories).

### Exposures

APOs were identified by delivery hospitalization records and included HDP (defined as pregnancy-associated hypertension, preeclampsia [formerly also called toxemia], and/or eclampsia), GD (yes, no), and PTB (birth at <37 weeks’ gestational age). Covariates included baseline city, mother’s age at birth, mother’s household income at baseline, whether the mother smoked cigarettes during pregnancy (yes, no), the primary caregiver’s highest educational attainment at any point across the entire study period (less than high school, high school or equivalent, some college, or college graduate or above), and offspring’s sex (male, female) and insurance status in young adulthood (insured, uninsured).

### CVH and Arterial Injury Definitions

Primary outcomes were clinical risk factor measures, including BMI, systolic blood pressure, diastolic blood pressure, HbA_1c_ level, total cholesterol level, and non–high-density lipoprotein (non-HDL) cholesterol level; CVH score, summarized by the American Heart Association’s LE8 measure; and arterial injury at study year 22.^16^ LE8 is a global assessment of CVH (score range, 0-100, with higher scores representing better CVH), calculated in this study from diet quality, physical activity, sleep, tobacco exposure, BMI, lipids, HbA_1c_ level, and blood pressure and also accounting for medication use for diabetes or hypertension.^16^ Continuous LE8 CVH scores were categorized as high (80-100), moderate (50-79), or low (0-49). A histogram of LE8 CVH scores is presented in the eFigure in Supplement 1.

Arterial injury measures were assessed via carotid ultrasonography. Mean-mean CIMT (in millimeters) was calculated as the mean of the right and left average mean CIMT of 3 measured R waves from an anterior approach and 3 from a posterior approach of the distal 1 cm of the common carotid artery far walls. Mean-maximum CIMT (in millimeters) was calculated as the mean of the right and left mean of the maximum CIMT of 3 measured R waves from an anterior approach and 3 from a posterior approach of the distal 1 cm of the common carotid artery far walls, the bulbs, and the proximal 1 cm of the internal carotid arteries. Both CIMT values are measures of subclinical arterial injury. Carotid grayscale median was calculated from the mean of the right and left average of 2 tracings from the distal 1 cm of the far wall of the common carotid arteries, with lower values representing higher cardiovascular risk.^20^ The distensibility coefficient was calculated as (*Ds*^2^ − *Dd*^2^)/(*PP* × *Dd*^2^), where *Ds* is the end-systolic diameter, *Dd* is the end-diastolic diameter, and *PP* is pulse pressure. The Young elastic modulus was calculated as (*Dd*/*h*)/*DC*, where *Dd* is the end-diastolic diameter, *h* is wall thickness, and *DC* is the distensibility coefficient.^21^ These are measurements of arterial stiffness.

### Statistical Analysis

Variable missingness is described in eTable 1 in Supplement 1. Missing variables were multiply imputed 25-fold using predictive mean matching via the mice package in R, version 4.3.0 (R Project for Statistical Computing).^22^ Effect estimates were pooled across the 25 imputed datasets using Rubin rules. Baseline enrollment and FF-CHAYA follow-up visit characteristics were described using means and SDs for continuous variables or frequencies and percentages for categorical variables. Differences in CVH measurements in young adulthood among participants exposed to each APO (vs not exposed) were estimated via Welch 2-sample *t* tests for continuous variables and χ^2^ tests for categorical variables. We used multivariable linear regression models to evaluate the associations of HDP or GD exposure or PTB, separately, with offspring’s BMI, systolic blood pressure, diastolic blood pressure, HbA_1c_ level, total cholesterol level, non-HDL cholesterol level, and continuous LE8 CVH score at the FF-CHAYA follow-up visit, adjusted for sociodemographic characteristics. We used multinomial logistic regression to assess associations of HDP or GD exposure or PTB, separately, with offspring’s year 22 categorical LE8 CVH score (low or moderate vs high [reference]), adjusted for sociodemographic factors. In addition, multivariable linear regression models were used to evaluate the associations of HDP or GD exposure or PTB, separately, with each offspring individual’s measure of arterial injury or stiffness, adjusted for sociodemographic characteristics. Model assumptions were assessed using standard diagnostic plots.

To minimize the potential confounding or intermediary role of impaired or excess fetal growth on offspring health in early adulthood, a sensitivity analysis was conducted among the subsample of participants who were born at appropriate weight for gestational age, defined as between the 10th and 90th percentiles of weight for gestational age.^23^ In this subsample of individuals born at weight appropriate for gestational age, the associations of APO exposure with offspring cardiovascular risk factors, LE8 CVH score, and arterial injury measures were evaluated.

For all analyses, statistical significance was set at 2-sided *P* < .05. Analyses were conducted from June 2024 to June 2025 in R, version 4.3.0.^24^

## Results

Of 4897 mother-child dyads in FFCWS, there were 1421 offspring participants in FF-CHAYA; 88 of these were ineligible for the current study due to missing key demographic data or mother’s pregnancy medical records indicating the presence of prepregnancy hypertension or diabetes or because the offspring individual was pregnant at the FF-CHAYA visit, resulting in a final analytic sample of 1333 participants. Mean (SD) offspring age was 22.4 (0.7) years at the FF-CHAYA year 22 follow-up visit. Of the 1333 participants in this study, 729 (55%) were female, 604 (45%) were male, 367 (28%) were Hispanic, 677 (51%) were Black, 230 (17%) were White, and 59 (4%) were other race and ethnicity. The mean (SD) maternal age at birth was 25.0 (6.0) years. Overall, 128 participants (10%) were exposed to HDP, 67 (5%) to GD, and 137 (10%) to PTB, and 43 (3%) had co-occurring APO exposure (18 [1%] with HDP and PTB, 10 [1%] with HDP and GD, 10 [1%] with GD and PTB, and 5 [<1%] with all 3 APOs). Additional demographic characteristics by APO exposure pooled over 25 imputed datasets are presented in Table 1, and characteristics among those exposed vs not exposed to individual APOs are presented in eTable 2 in Supplement 1. The distribution of offspring by baseline city is presented in eTable 3 in Supplement 1. Participants with HDP exposure had primary caregivers (mothers, for 1276 participants [96%]) with lower educational attainment than the caregivers of participants without exposure to HDP. Participants exposed to GD were born to mothers who were older on average at time of delivery. Participants with PTB were more often born to mothers who smoked cigarettes during pregnancy.

**Table 1.** Offspring Characteristics by Adverse Pregnancy Outcome Exposure

Characteristic	Participants^a^				
	HDP (n = 128)	GD (n = 67)	PTB (n = 137)	No APOs (n = 1048)	Overall (N = 1333)
**Baseline**					
Sex					
Female	74 (58)	34 (51)	73 (53)	574 (55)	729 (55)
Male	54 (42)	33 (49)	64 (47)	474 (45)	604 (45)
Race and ethnicity					
Hispanic	25 (20)	19 (28)	37 (27)	300 (29)	367 (28)
Non-Hispanic Black	79 (62)	32 (48)	73 (53)	521 (50)	677 (51)
Non-Hispanic White	20 (16)	8 (12)	24 (18)	181 (17)	230 (17)
Other^b^	4 (3)	8 (12)	3 (2)	46 (4)	59 (4)
Mother’s annual family income, $	30 124 (31 440)	30 165 (30 288)	33 615 (31 712)	33 921 (33 562)	33 526 (33 215)
Primary caregiver’s highest educational attainment					
<HS	32 (25)	14 (21)	20 (15)	152 (15)	206 (16)
HS or equivalent	57 (45)	28 (42)	63 (46)	441 (42)	565 (42)
Some college	25 (20)	10 (15)	27 (20)	218 (21)	272 (20)
≥College graduate	14 (11)	15 (22)	27 (20)	237 (23)	290 (22)
Mother’s age at birth, y	25 (6)	30 (7)	26 (6)	25 (6)	25 (6)
Mother smoked cigarettes during pregnancy	22 (17)	8 (12)	38 (28)	215 (21)	277 (21)
**Follow-up (year 22)**					
Had health insurance	101 (79)	56 (84)	116 (85)	851 (81)	1086 (81)
BMI	32 (9)	29 (8)	28 (9)	29 (8)	29 (8)
Waist circumference, cm	87 (27)	79 (23)	84 (23)	82 (23)	83 (23)
Random glucose, mg/dL	100 (41)	101 (37)	103 (53)	92 (15)	94 (23)
HbA_1c_, %	5.5 (1.1)	5.4 (0.9)	5.6 (1.3)	5.3 (0.4)	5.3 (0.6)
Blood pressure, mm Hg					
Systolic	117 (11)	118 (12)	116 (11)	115 (12)	116 (12)
Diastolic	72 (10)	71 (10)	69 (9)	69 (10)	69 (10)
Cholesterol, mg/dL					
Total	164 (30)	165 (34)	164 (34)	165 (33)	165 (33)
LDL	96 (27)	95 (29)	95 (30)	95 (30)	95 (30)
HDL	49 (16)	50 (14)	50 (15)	52 (15)	51 (15)
Triglycerides, mg/dL	94 (50)	98 (64)	100 (73)	94 (59)	94 (60)
Creatinine, mg/dL	0.79 (0.19)	0.80 (0.18)	0.83 (0.20)	0.82 (0.35)	0.82 (0.32)
Carotid artery measures					
IMT, mm					
Maximum	0.66 (0.07)	0.66 (0.06)	0.64 (0.07)	0.64 (0.06)	0.64 (0.07)
Mean	0.52 (0.07)	0.51 (0.06)	0.50 (0.06)	0.50 (0.06)	0.50 (0.06)
Grayscale median	81 (14)	82 (14)	83 (13)	84 (13)	84 (13)
Distensibility coefficient	0.01 (0.00)	0.01 (0.00)	0.01 (0.00)	0.01 (0.00)	0.01 (0.00)
Young elastic modulus	898 (396)	1056 (642)	911 (397)	928 (430)	928 (434)
LE8 score					
CVH	67 (13)	71 (13)	69 (14)	69 (13)	69 (13)
Category					
High (80-100)	20 (16)	21 (31)	33 (24)	235 (22)	299 (22)
Moderate (50-79)	97 (76)	43 (64)	93 (68)	736 (70)	937 (70)
Low (0-49)	11 (9)	3 (4)	11 (8)	77 (7)	96 (7)

Abbreviations: APO, adverse pregnancy outcome; BMI, body mass index (calculated as weight in kilograms divided by height in meters squared); BP, blood pressure; CVH, cardiovascular health; GD, gestational diabetes; HbA_1c_, glycated hemoglobin; HDL, high-density lipoprotein; HDP, hypertensive disorders of pregnancy; HS, high school; IMT, intima-media thickness; LDL, low-density lipoprotein; LE8, Life’s Essential 8; PTB, preterm birth.

SI conversion factors: To convert cholesterol (total, HDL, and LDL) to mmol/L, multiply by 0.0259; creatinine to µmol/L, multiply by 88.4; glucose to mmol/L, multiply by 0.0555; HbA_1c_ to proportion of total hemoglobin, multiply by 0.01; triglycerides to mmol/L, multiply by 0.0113.

^a^ Data are presented as mean (SD) or median (IQR) for continuous variables or number (percentage) of participants for categorical variables.

^b^ Indicates multiracial or any other self-reported category.

In adjusted analyses, exposure to HDP was associated with higher BMI (adjusted β, 2.80; 95% CI, 1.07-4.53), higher diastolic blood pressure (adjusted β, 2.29; 95% CI, 0.17-4.41), and higher HbA_1c_ level (adjusted β, 0.21; 95% CI, 0.02-0.41) among offspring in young adulthood (Table 2). PTB was associated with higher HbA_1c_ level (adjusted β, 0.29; 95% CI, 0.15-0.43). There were no associations between GD exposure and clinical risk factors. The associations of APOs with LE8 CVH scores are shown in Table 3 and Table 4. Exposure to HDP was not associated with overall LE8 CVH score but was associated with a higher component LE8 score for sleep (adjusted β, 7.65; 95% CI, 2.04-13.25) and lower component LE8 scores for BMI (adjusted β, −11.24; 95% CI, −19.09 to −3.40), glucose level (adjusted β, −5.27; 95% CI, −9.28 to −1.26), and blood pressure (adjusted β, −5.26; 95% CI, −9.96 to −0.55). GD exposure was associated with a lower LE8 component score for blood pressure (adjusted β, −6.59; 95% CI, −13.95 to −0.16). PTB exposure was associated with a lower LE8 component score for glucose (adjusted β, −3.73; 95% CI, −7.22 to −0.23).

**Table 2.** Association of APOs With Offspring Cardiovascular Risk Factors

Risk factor	Adjusted β (95% CI) for APO exposure vs no exposure^a^		
	HDP	GD	PTB
BMI	2.80 (1.07 to 4.53)^b^	0.91 (−1.33 to 3.15)	−0.63 (−2.20 to 0.93)
Blood pressure			
Systolic	1.61 (−0.69 to 3.91)	2.90 (−0.41 to 6.21)	0.71 (−1.46 to 2.88)
Diastolic	2.29 (0.17 to 4.41)^b^	2.30 (−0.94 to 5.55)	0.03 (−2.00 to 2.05)
HbA_1c_	0.21 (0.02 to 0.41)^b^	0.14 (−0.18 to 0.45)	0.29 (0.15 to 0.43)^b^
Cholesterol			
Total	−1.52 (−8.76 to 5.72)	−0.27 (−10.65 to 10.10)	−0.54 (−7.92 to 6.84)
Non-HDL	0.73 (−7.00 to 8.45)	0.91 (−9.81 to 11.63)	0.33 (−7.12 to 7.78)

Abbreviations: APO, adverse pregnancy outcome; BMI, body mass index; GD, gestational diabetes; HbA_1C_, glycated hemoglobin; HDL, high-density lipoprotein; HDP, hypertensive disorders of pregnancy; PTB, preterm birth.

^a^ Multivariable linear regression, adjusted for baseline city, mother’s age at birth, household income at baseline, primary caregiver’s highest educational attainment at any point across the study period, mother’s smoking cigarettes during pregnancy, offspring’s insurance status at year 22, and offspring’s sex.

^b^ *P* < .05.

**Table 3.** Association of APOs With LE8 CVH Scores Among Offspring

LE8 score category	Adjusted β (95% CI) for APO exposure vs no exposure^a^		
	HDP	GD	PTB
Overall CVH	−2.10 (−4.70 to 0.49)	1.08 (−2.61 to 4.77)	0.19 (−2.54 to 2.92)
Diet	−3.15 (−9.61 to 3.31)	3.13 (−5.69 to 11.94)	−1.64 (−7.26 to 4.97)
Physical activity	−5.33 (−15.40 to 4.73)	10.75 (−2.23 to 23.73)	3.70 (−6.50 to 13.90)
Tobacco	5.70 (−2.56 to 13.95)	6.78 (−3.71 to 17.28)	3.49 (−4.54 to 11.51)
Sleep	7.65 (2.04 to 13.25)^b^	0.30 (−7.48 to 8.08)	−2.83 (−8.39 to 2.72)
BMI	−11.24 (−19.09 to −3.40)^b^	−1.45 (−11.69 to 8.79)	3.88 (−3.38 to 11.15)
Cholesterol	−0.09 (−5.05 to 5.22)	−0.66 (−8.23 to 6.91)	−2.05 (−7.16 to 3.07)
Glucose	−5.27 (−9.28 to −1.26)^b^	−3.61 (−9.90 to 2.68)	−3.73 (−7.22 to −0.23)^b^
Blood pressure	−5.26 (−9.96 to −0.55)^b^	−6.59 (−13.95 to −0.16)^b^	0.70 (−3.98 to 5.38)

Abbreviations: APO, adverse pregnancy outcome; BMI, body mass index; CVH, cardiovascular health; GD, gestational diabetes; HDP, hypertensive disorders of pregnancy; LE8, Life’s Essential 8; PTB, preterm birth.

^a^ Adjusted for baseline city, mother’s age at birth, household income at baseline, primary caregiver’s highest educational attainment at any point across the study period, mother’s smoking cigarettes during pregnancy, offspring’s insurance status at year 22, and offspring’s sex. LE8 score (range, 0-100, with higher scores indicating better cardiovascular health) was measured using a composite score of cholesterol and glucose levels, blood pressure, BMI, smoking, diet quality, physical activity, and sleep.

^b^ *P* < .05.

**Table 4.** AORs for Association of APOs With Lower LE8 Scores in Young Adulthood

LE8 overall CVH score	AOR (95% CI) by presence vs absence of APOs^a^		
	HDP	GD	PTB
High (80-100)	1 [Reference]	1 [Reference]	1 [Reference]
Moderate (50-79)	1.51 (0.86-2.67)	0.73 (0.37-1.44)	0.86 (0.51-1.45)
Low (0-49)	1.59 (0.64-3.97)	0.41 (0.06-2.61)	1.00 (0.40-2.54)

Abbreviations: AOR, adjusted odds ratio; APO, adverse pregnancy outcome; CVH, cardiovascular health; HDP, hypertensive disorders of pregnancy; GD, gestational diabetes; LE8, Life’s Essential 8; PTB, preterm birth.

^a^ Adjusted for baseline city, mother’s age at birth, household income at baseline, primary caregiver’s highest educational attainment at any point across the study period, mother’s smoking cigarettes during pregnancy, offspring’s insurance status at year 22, and offspring’s sex.

The associations of exposure to APOs with arterial injury measures in young adulthood are shown in Table 5. Exposure to HDP was associated with higher maximum CIMT (adjusted β, 0.02; 95% CI, 0.01-0.04), higher mean CIMT (adjusted β, 0.02; 95% CI, 0.01-0.03), and lower carotid grayscale median (adjusted β, −3.68; 95% CI, −6.30 to −1.05). Exposure to GD was associated with higher maximum CIMT (adjusted β, 0.02; 95% CI, 0.00-0.04) and mean CIMT (adjusted β, 0.02; 95% CI, 0.00-0.04). There were no associations of PTB with measures of early arterial injury.

**Table 5.** Association of APOs With Offspring Early Arterial Injury in Young Adulthood

Arterial measure	Adjusted β (95% CI) for APO exposure vs no exposure^a^		
	HDP	GD	PTB
Common carotid IMT			
Maximum	0.02 (0.01 to 0.04)	0.02 (0.00 to 0.04)	0.00 (−0.01 to 0.02)
Mean	0.02 (0.01 to 0.03)	0.02 (0.00 to 0.04)	0.00 (−0.02 to 0.01)
Carotid grayscale median	−3.68 (−6.30 to −1.05)	−1.56 (−5.25 to 2.12)	−0.56 (−3.29 to 2.17)
Distensibility coefficient	0.00 (0.00 to 0.00)	0.00 (0.00 to 0.00)	0.00 (0.00 to 0.00)
Young elastic modulus	−14.57 (−105.38 to 76.25)	116.87 (−26.71 to 260.46)	−20.62 (−108.21 to 66.97)

Abbreviations: APO, adverse pregnancy outcome; GD, gestational diabetes; HDP, hypertensive disorders of pregnancy; IMT, intima-media thickness; PTB, preterm birth.

^a^ Multivariable linear regression adjusted for baseline city, mother’s age at birth, household income at baseline, primary caregiver’s highest educational attainment at any point across the study period, mother smoking cigarettes during pregnancy, offspring’s insurance status at year 22, and offspring’s sex.

In sensitivity analyses restricting the sample to only those born at weight appropriate for gestational age (n = 1155), associations of HDP exposure with cardiovascular risk factors, component LE8 scores, and arterial injury measures and association of PTB with HbA_1c_ levels remained generally consistent and significant (eTables 4-6 in Supplement 1). In the limited subsample, exposure to HDP was additionally associated with lower overall LE8 CVH score (adjusted β, −3.80; 95% CI, −6.93 to −0.66) in early adulthood. The associations of GD exposure with LE8 blood pressure score, maximum CIMT, and mean CIMT were attenuated.

## Discussion

In this longitudinal prospective cohort study, HDP exposure in utero was associated with multiple cardiometabolic risk factors in early adulthood, including higher BMI, higher diastolic blood pressure, and higher HbA_1c_ level, and with early arterial injury. GD exposure was associated with a worse LE8 blood pressure score and early arterial injury, and PTB was associated with higher HbA_1c_ levels. Furthermore, in sensitivity analyses of offspring born at weight appropriate for gestational age, associations of HDP exposure with clinical risk factors, LE8 scores, and arterial injury and the association of PTB with higher HbA_1c_ levels remained robust, indicating that associations were independent of impaired or excessive fetal growth. The associations of GD exposure with blood pressure score and early arterial injury were attenuated in sensitivity analyses, suggesting that these associations may have been mediated in part by excess fetal growth among offspring. Overall, these findings suggest that in utero fetal exposure to APOs may be associated with cardiovascular and metabolic health of offspring longitudinally into early adulthood.

A growing body of research supports the importance of maternal health for offspring health.^7,25,26^ A systematic review found that gestational hypertension was consistently associated with higher blood pressure among offspring, but associations were mixed for preeclampsia.^27^ A prior study using data from a European national registry found that exposure to HDP was associated with 1.5-times higher risk of death due to cardiovascular disease among offspring.^28^ Our findings extend prior analyses by evaluating the association between maternal health and offspring CVH in young adulthood, approximately 10 years beyond the offspring age evaluated in the HAPO study.^15^ Our study further adds to the literature by leveraging objective carotid artery measures to detect early arterial injury. Assessment of these outcomes captures a critical life-course stage for intervention, when cardiovascular risk trajectories are often established but overt clinical disease has not yet developed.

In our analysis, HDP exposure during gestation was significantly associated with arterial injury. The current study found a 0.02-mm higher maximum CIMT among those with vs without HDP exposure, which likely in part reflects greater precision with a larger sample. In young adults, this difference in CIMT is equivalent to approximately 3 to 5 years of vascular aging.^29^ In general, a 0.1-mm increase in CIMT is associated with a 34% higher risk of myocardial infarction, stroke, or mortality among younger adults.^30^ Endothelial dysfunction, observed among individuals exposed to HDP,^31^ may lead to inferior vascular function, potentially explaining the higher CIMT and lower carotid grayscale median among young adults with HDP exposure in this study.

In the current study, GD was associated with a lower LE8 blood pressure score and increased maximum CIMT and mean CIMT. Prior studies found that exposure to GD was associated with a higher BMI *z* score^32^ and impaired glucose tolerance^33^ in early childhood. A prior study found no associations between GD and offspring CIMT,^34^ though our study provided 16 additional years of follow-up and a greater sample size. At the time of the FFCWS enrollment visit (1998-2000), guidelines recommended screening for GD in high-prevalence populations and patients with risk factors, but universal screening was not routine clinical practice.^35,36^ It is possible that cases of GD were missed in the current study’s cohort, resulting in nondifferential misclassification and attenuation of results to the null.^37,38^

Our analysis identified a significant association between PTB and higher HbA_1c_ levels among offspring in young adulthood. Meta-analyses have reported higher systolic blood pressure,^39^ fat mass, diastolic blood pressure, fasting glucose level, insulin level, and total cholesterol level in adult offspring born preterm compared with those born at 37 or more weeks’ gestation.^40^ However, these studies used between-group comparisons rather than regression models, without adjustment for confounding variables. Our study did not observe associations between PTB and arterial injury, consistent with prior research that did not find differences in CIMT dimensions in offspring born preterm compared with those born at 37 or more weeks’ gestation.^41^

The associations between exposure to APOs and cardiometabolic risk factors in early adulthood may be mediated through several mechanisms, including shared genetics, health behaviors, social determinants of health, and biological pathways, including oxidative stress and inflammatory pathways. Genes and lifestyle behaviors that predispose individuals to worse CVH might be transmitted and shared across generations to cause the APOs among mothers and arterial injury among offspring observed in this study. APO exposure during gestation may be associated with offspring CVH through changes to uterine environment, placental function, epigenetics, and gut microbiota.^42^ For example, in spontaneous mouse models of preeclampsia, placental abnormalities in utero^43^ and higher blood pressure and cardiomegaly among female offspring in early adulthood have been observed.^44^ Research on pregnancies with GD reveals changes to the maternal microbiome,^45^ and offspring exposed to GD show reductions in microbial diversity that may be associated with higher risk of metabolic diseases.^46^

This study has several clinical and public health implications. Evidence of worse clinical risk factor levels, lower LE8 CVH scores, and early arterial injury among participants with exposure to HDP during gestation represent early and potentially intervenable development of risk factors for future cardiovascular disease events. Our results suggest that suboptimal CVH may be intergenerational and that transmission may occur via pathways beyond altered fetal growth. At birth, individuals may be disadvantaged regarding lifetime CVH based on their exposure to APOs and shared environments. Interventions before and during pregnancy are needed to promote both maternal and offspring health across the life course.^47^ Future work is needed to delineate the mechanisms by which various APOs act to explain the differential associations of CVH with arterial injury.

### Strengths and Limitations

Strengths of this analysis include a large, diverse participant sample enrolled from 20 US cities. The longitudinal cohort design with over 2 decades of follow-up supports the evaluation of HDP as an intergenerational risk factor for arterial injury and suboptimal CVH in offspring. In addition, APO status was abstracted from pregnancy records rather than self-reported, and objective measures for arterial injury and a variety of objective CVH measures were collected.

Several limitations are also noted. First, the prevalence of APOs was low, in part because the FFCWS study generally did not recruit mothers who experienced peripartum complications or whose newborns experienced complications.^18^ Second, the observational nature of the dataset precludes causal inference, although the prospective, longitudinal nature of the data supports the potential role of APOs in health in early adulthood. Third, the FFCWS study enrolled individuals from 1998 to 2000. Diagnostic guidelines have since changed, which may have led to underrecognition of APOs (particularly GD). Fourth, we did not apply formal correction for multiple hypothesis testing, and some findings may therefore be subject to an increased risk of type I error. Fifth, small sample sizes for offspring exposed to each APO likely limited power to detect some associations and precluded our ability to stratify the sample by offspring sex or race and ethnicity. Future research should explore how associations between in utero exposure to APOs and CVH in early adulthood may vary by experiences of adverse social conditions, given the differences in social risk factors that may contribute to higher risk for APOs and suboptimal CVH in persons giving birth and their offspring.

## Conclusions

In this longitudinal cohort study of mother-child dyads followed through young adulthood among offspring, exposure to APOs, particularly HDP, was associated with worse cardiovascular risk factors and early arterial injury in early adulthood. These findings may portend a higher future risk for adverse cardiovascular outcomes among offspring exposed to APOs. Future research must delineate the roles that various APOs may have in association with offspring CVH and whether differences exist by offspring sex or whether associations are modified by social risk factors. Interventions to improve maternal CVH before and during pregnancy may contribute to better offspring CVH across the life course.

## Notes

Supplement 1. **eTable 1.** Missing Data Summary by Variable **eTable 2.** Offspring Characteristics at Year 22 by Adverse Pregnancy Outcome Exposure vs No Exposure **eTable 3.** Distribution of Offspring by Baseline City **eTable 4.** Sensitivity Analysis of Offspring Born at Weight Appropriate for Gestational Age: Cardiovascular Risk Factors **eTable 5.** Sensitivity Analysis of Offspring Born at Weight Appropriate for Gestational Age: Life’s Essential 8 Cardiovascular Health Scores **eTable 6.** Sensitivity Analysis of Offspring Born at Weight Appropriate for Gestational Age: Early Arterial Injury **eFigure.** Histogram of Life’s Essential 8 Cardiovascular Health Scores

Supplement 2.
Data Sharing Statement
