# Supplementary material for: Adverse Pregnancy Outcomes and Cardiovascular Health Among Offspring in Early Adulthood
Source: JAMA Netw Open. 2026 May 14;9(5):e266783. doi: 10.1001/jamanetworkopen.2026.6783 (PMC13177028; doi:10.1001/jamanetworkopen.2026.6783)
Supplement: Supplement 1. — eTable 1. Missing Data Summary by Variable eTable 2. Offspring Characteristics at Year 22 by Adverse Pregnancy Outcome Exposure vs No Exposure eTable 3. Distribution of Offspring by Baseline City eTable 4. Sensitivity Analysis of Offspring Born at Weight Appropriate for Gestational Age: Cardiovascular Risk Factors eTable 5. Sensitivity Analysis of Offspring Born at Weight Appropriate for Gestational Age: Life’s Essential 8 Cardiovascular Health Scores eTable 6. Sensitivity Analysis of Offspring Born at Weight Appropriate for Gestational Age: Early Arterial Injury eFigure. Histogram of Life’s Essential 8 Cardiovascular Health Scores [file jamanetwopen-e266783-s001.pdf]

Supplementary Online Content

Lam EL, Gauen AM, Khan SS, et al. Adverse pregnancy outcomes and cardiovascular health among offspring in early adulthood. *JAMA Netw Open*. 2026;9(5):e266783. doi:10.1001/jamanetworkopen.2026.6783

eTable 1. Missing Data Summary by Variable

eTable 2. Offspring Characteristics at Year 22 by Adverse Pregnancy Outcome Exposure vs No Exposure

eTable 3. Distribution of Offspring by Baseline City

eTable 4. Sensitivity Analysis of Offspring Born at Weight Appropriate for Gestational Age: Cardiovascular Risk Factors

eTable 5. Sensitivity Analysis of Offspring Born at Weight Appropriate for Gestational Age: Life's Essential 8 Cardiovascular Health Scores

eTable 6. Sensitivity Analysis of Offspring Born at Weight Appropriate for Gestational Age: Early Arterial Injury

eFigure. Histogram of Life's Essential 8 Cardiovascular Health Scores

This supplementary material has been provided by the authors to give readers additional information about their work.

© 2026 Lam EL et al. *JAMA Network Open*.

eTable 1. Missing Data Summary by Variable

	Missing (n)	% Missing
Birthweight (grams)	327	24.5
Gestational Age	320	24.0
Preterm birth	320	24.0
Smoking during pregnancy	318	23.9
Hypertensive disorders of pregnancy	318	23.9
Gestational diabetes	318	23.9
PCG's highest educational attainment	164	12.3
Hemoglobin A1c (%)	99	7.4
Fasting glucose (mg/dL)	97	7.3
Total cholesterol (mg/dL)	97	7.3
LDL cholesterol (mg/dL)	97	7.3
HDL cholesterol (mg/dL)	97	7.3
Non-HDL cholesterol (mg/dL)	97	7.3
Triglycerides (mg/dL)	97	7.3
Creatinine (mg/dL)	97	7.3
Glucose LE8	72	5.4
Non-HDL-C LE8	71	5.3
Physical Activity LE8	62	4.7
Sleep LE8	43	3.2
Distensibility coefficient, mean (SD)	38	2.9
Young's elastic modulus, mean (SD)	38	2.9
BMI LE8	21	1.6
BMI (kg/m^2)	21	1.6
Insurance status	19	1.4
Common grayscale median, mean (SD)	14	1.1
Diet LE8	10	0.8
Waist circumference (cm)	9	0.7
Low birthweight	8	0.6
Mean-mean cIMT, mean (SD)	7	0.5
Mean-max cIMT, mean (SD)	7	0.5
Participant age at year 22	3	0.2
Smoking LE8	2	0.2
Participant race and ethnicity	2	0.2
Mother's baseline age	1	0.1

BMI: bodym mass index, cIMT: carotid intima-media thickness, HDL: High-density lipoprotein, LDL: low-density lipoprotein, LE8: Life's Essential 8, PCG: Primary caregiver, SD: Standard deviation

© 2026 Lam EL et al. *JAMA Network Open*.

eTable 2. Offspring Characteristics at Year 22 by Adverse Pregnancy Outcome Exposure vs no Exposure

	HDP			GDM			PTB		
	**No HDP (n=1205)**	**HDP (n=128)**	**p-value[a]**	**No GDM (n=1266)**	**GDM (n=67)**	**p-value[a]**	**No PTB (n=1196)**	**PTB (n=137)**	**p-value[a]**
Baseline									
Female, n (%)	656 (54%)	74 (58%)	0.54	696 (55%)	34 (51%)	0.60	657 (55%)	73 (53%)	0.72
Race/ethnic group, n (%)			0.10			0.23			0.59
Hispanic	341 (28%)	25 (20%)		349 (28%)	19 (28%)		332 (28%)	37 (27%)	
Non-Hispanic Black	598 (50%)	79 (62%)		645 (51%)	32 (48%)		604 (50%)	73 (53%)	
Non-Hispanic White	211 (18%)	20 (16%)		221 (17%)	8 (12%)		203 (17%)	24 (18%)	
Other[b]	55 (4%)	4 (3%)		51 (4%)	8 (12%)		57 (5%)	3 (2%)	
Mother's household income, mean (SD)	$33,885 (33,390)	$30,124 (31 440)	0.32	$33,702 (33,363)	$30,165 (30,288))	0.49	$33,517 (33,400)	$33,615 (31,712)	>0.9
Primary care giver's highest educational attainment, n (%)			0.01			0.54			0.85
< HS	174 (14%)	32 (25%)		191 (15%)	14 (21%)		187 (16%)	20 (15%)	
HS or equivalent	510 (42%)	57 (45%)		539 (43%)	28 (42%)		503 (42%)	63 (46%)	
Some college	246 (20%)	25 (20%)		261 (21%)	10 (15%)		244 (20%)	27 (20%)	
≥ College	275 (23%)	14 (11%)		275 (22%)	15 (22%)		262 (22%)	27 (20%)	
Mother's age, mean (SD)	25 (6)	25 (6)	0.33	25 (6)	30 (7)	<0.001	25 (6)	26 (6)	0.25
Mother smoking during pregnancy, n (%)	253 (21%)	22 (17%)	0.43	267 (21%)	8 (12%)	0.10	237 (20%)	38 (28%)	0.06
Year 22									
Insurance, n (%)	987 (82%)	101 (79%)	0.46	1030 (81%)	56 (84%)	0.74	970 (81%)	116 (85%)	0.39
BMI, mean (SD)	29 (8)	32 (9)	<0.001	29 (8)	29 (8)	0.70	29 (8)	28 (9)	0.44
Waist circumference, mean (SD)	82 (23)	87 (27)	0.06	83 (24)	79 (23)	0.26	83 (24)	84 (23)	0.48
Random glucose, mean (SD)	93 (20)	100 (41)	0.02	94 (22)	101 (37)	0.15	93 (17)	103 (53)	<0.001
Hemoglobin A1c, mean (SD)	5.3 (0.5)	5.5 (1.1)	0.03	5.3 (0.6)	5.4 (0.9)	0.38	5.3 (0.5)	5.6 (1.3)	<0.001
Systolic blood pressure, mean (SD)	116 (12)	117 (11)	0.21	116 (12)	118 (12)	0.12	116 (12)	116 (11)	0.48
Diastolic blood pressure, mean (SD)	69 (10)	72 (10)	0.01	69 (10)	71 (10)	0.27	69 (10)	69 (9)	>0.9
Total Cholesterol, mean (SD)	165 (33)	164 (30)	0.77	165 (33)	165 (34)	>0.9	165 (33)	164 (34)	>0.9
LDL cholesterol, mean (SD)	95 (30)	96 (27)	0.79	95 (30)	95 (29)	>0.9	95 (30)	95 (30)	>0.9
HDL cholesterol, mean (SD)	52 (15)	49 (16)	0.25	52 (15)	50 (14)	0.63	52 (15)	50 (15)	0.45
Triglycerides, mean (SD)	94 (61)	94 (50)	>0.9	94 (60)	98 (64)	0.74	94 (59)	100 (73)	0.44
Creatinine, mean (SD)	0.8 (0. 3)	0.79 (0.2)	0.27	0.8 (0.3)	0.8 (0.2)	0.61	0.8 (0.3)	0.8 (0.2)	0.54
Carotid artery measures, mean (SD)									
Maximum IMT, mm	0.64 (0.07)	0.66 (0.07)	0.004	0.64 (0.07)	0.66 (0.06)	0.12	0.64 (0.07)	0.64 (0.07)	0.57
Mean IMT, mm	0.50 (0.06)	0.52 (0.07)	0.006	0.50 (0.06)	0.51 (0.06)	0.16	0.50 (0.06)	0.50 (0.06)	0.70
Grayscale median	84 (13)	81 (14)	0.01	84 (13)	82 (14)	0.29	84 (13)	83 (13)	0.65
Distensibility coefficient	0.007 (0.002)	0.007 (0.002)	>0.9	0.007 (0.002)	0.007 (0.003)	0.20	0.007 (0.002)	0.007 (0.002)	0.87
Young's elastic modulus	931 (438)	898 (396)	0.47	921 (419)	1056 (642)	0.08	930 (438)	911 (397)	0.71
Life's Essential 8 CVH score, mean (SD)	69 (13)	67 (13)	0.047	69 (13)	71 (13)	0.24	69 (13)	69 (14)	>0.9

© 2026 Lam EL et al. *JAMA Network Open*.

Categorical LE8 CVH score, n (%)			0.39			0.54			>0.9
High (score 80-100)	278 (23%)	20 (16%)		278 (22%)	21 (31%)		265 (22%)	33 (24%)	
Moderate (score 50-79)	841 (70%)	97 (76%)		894 (71%)	43 (64%)		845 (71%)	93 (68%)	
Low (score 0-49)	86 (7%)	11 (9%)		94 (7%)	3 (5%)		86 (7%)	11 (8%)	

Abbreviations: APO, adverse pregnancy outcome; HDP, hypertensive disorders of pregnancy; GDM, gestational diabetes mellitus; PTB, pre-term birth; HS, high school; BMI, body mass index; IMT, intima-media thickness; CVH, cardiovascular health; LE8, Life's Essential 8.

[a]Pearson's Chi-squared test or Welch Two Sample t-test

[b]Other race indicates Multiracial or Other

© 2026 Lam EL et al. *JAMA Network Open*.

eTable 3. Distribution of Offspring by Baseline City

Baseline City	n (%)[a]
Austin	83 (6.2%)
Baltimore	78 (5.9%)
Boston	33 (2.5%)
Chicago	43 (3.2%)
Corpus Christi	103 (7.7%)
Detroit	119 (8.9%)
Indianapolis	109 (8.2%)
Jacksonville	26 (2.0%)
Milwaukee	128 (9.6%)
Nashville	28 (2.1%)
New York	56 (4.2%)
Newark	93 (7.0%)
Norfolk	32 (2.4%)
Oakland	84 (6.3%)
Philadelphia	93 (7.0%)
Pittsburgh	27 (2.0%)
Richmond	59 (4.4%)
San Antonio	25 (1.9%)
San Jose	86 (6.5%)
Toledo	28 (2.1%)

[a]Frequencies stratified by APO status are not provided to maintain participant confidentiality due to relatively low samples of participants exposed to APOs.

© 2026 Lam EL et al. *JAMA Network Open*.

eTable 4. Sensitivity Analysis of Offspring Born at Weight Appropriate for Gestational Age: Cardiovascular Risk Factors

	Adjusted β (95% CI) for APO exposure (versus no exposure)		
	**HDP**	**GDM**	**PTB**
BMI, kg/m^2	**3.52 (1.50, 5.54)**	1.16 (-1.70, 4.02)	-1.65 (-3.64, 0.33)
Systolic blood pressure, mmHg	1.11 (-1.57, 3.79)	2.34 (-1.41, 6.10)	0.70 (-1.93, 3.32)
Diastolic blood pressure, mmHg	**2.28 (0.03, 4.79)**	1.32 (-2.21, 4.85)	-0.94 (-3.40, 1.52)
HbA1c, %	**0.19 (0.08, 0.31)**	0.12 (-0.05, 0.29)	**0.11 (0.00, 0.23)**
Total cholesterol, mg/dL	-2.59 (-11.00, 5.81)	1.05 (-10.40, 12.50)	-2.12 (-10.28, 6.04)
Non-HDL cholesterol, mg/dL	0.20 (-8.40, 8.80)	2.53 (-9.33, 14.38)	-2.84 (-11.24, 5.56)

Bold indicates statistically significant at p<0.05. Abbreviations: APO, adverse pregnancy outcome; CI, confidence interval; HDP, hypertensive disorders of pregnancy; GDM, gestational diabetes mellitus; PTB, pre-term birth; BMI, body mass index; HDL, high-density lipoprotein. Multivariable linear regression adjusted for baseline city, mother's age at birth, household income at baseline, primary caregiver's highest educational attainment at any point across the study period, mother smoking during pregnancy, offspring's insurance status at year 22, and offspring's sex.

© 2026 Lam EL et al. *JAMA Network Open*.

eTable 5. Sensitivity Analysis of Offspring Born at Weight Appropriate for Gestational Age: Life's Essential 8 Cardiovascular Health Scores

	**Adjusted β (95% CI) for APO exposure (versus no exposure)**		
	**HDP**	**GDM**	**PTB**
Overall LE8 CVH Score	**-3.80 (-6.93, -0.66)**	0.99 (-3.46, 5.45)	0.61 (-2.51, 3.73)
LE8 diet score	-2.98 (-10.53, 4.56)	5.85 (-4.65, 16.36)	-5.40 (-12.73, 1.92)
LE8 physical activity score	-9.46 (-20.87, 1.94)	13.91 (-2.67, 30.49)	3.16 (-8.58, 14.90)
LE8 tobacco score	0.64 (-8.75, 10.03)	3.61 (-9.57, 16.79)	1.86 (-7.33, 11.06)
LE8 sleep score	**7.72 (1.21, 14.23)**	-3.28 (-12.37, 5.81)	-3.79 (-10.13, 2.55)
LE8 BMI score	**-14.08 (-22.96, -5.20)**	-3.37 (-15.96, 9.22)	9.59 (0.90, 18.29)
LE8 cholesterol score	-0.6 (-6.42, 5.22)	-0.80 (-8.89, 7.29)	-0.41 (-6.09, 5.26)
LE8 glucose score	**-5.68 (-9.31, -2.05)**	-3.29 (-8.38, 1.81)	-1.38 (-4.93, 2.17)
LE8 blood pressure score	**-5.92 (-11.61, -0.23)**	-4.68 (-12.67, 3.32)	1.26 (-4.33, 6.84)
	**Odds ratio (95% CI) associated with presence (versus absence) of APO**		
	**HDP**	**GDM**	**PTB**
High CVH (score 80-100)	*Reference*	*Reference*	*Reference*
Moderate CVH (score 50-79)	1.75 (0.88, 3.51)	0.63 (0.29, 1.36)	0.80 (0.45, 1.44)
Low CVH (score 0-49)	2.43 (0.86, 6.91)	0.42 (0.06, 2.91)	0.78 (0.27, 2.29)

Bold indicates statistically significant at p<0.05. Abbreviations: APO, adverse pregnancy outcome; CI, confidence interval; HDP, hypertensive disorders of pregnancy; GDM, gestational diabetes mellitus; PTB, pre-term birth; LE8, Life's Essential 8; CVH, cardiovascular health; BMI, body mass index. Multivariable linear or multinomial logistic regression adjusted for baseline city, mother's age at birth, household income at baseline, primary caregiver's highest educational attainment at any point across the study period, mother smoking during pregnancy, offspring's insurance status at year 22, and offspring's sex. LE8 score (0-100) was measured using a composite score of cholesterol and glucose levels, blood pressure, body mass index, smoking, diet quality, physical activity, and sleep.

© 2026 Lam EL et al. *JAMA Network Open*.

eTable 6. Sensitivity Analysis of Offspring Born at Weight Appropriate for Gestational Age: Early Arterial injury

	**Adjusted β (95% CI) for APO exposure (versus no exposure)**		
	**HDP**	**GDM**	**PTB**
Maximum common carotid IMT, mm	**0.015 (0.001, 0.030)**	0.018 (-0.003, 0.039)	-0.006 (-0.020, 0.009)
Mean common carotid IMT, mm	0.014 (0.000, 0.028)	0.011 (-0.009, 0.031)	-0.013 (-0.027, 0.001)
Grayscale median	**-4.88 (-8.05, -1.72)**	-1.02 (-5.48, 3.44)	-0.25 (-3.37, 2.88)
Distensibility coefficient	0.0000 (-0.0005, 0.0005)	-0.0002 (-0.0009, 0.0006)	-0.0001 (-0.0006, 0.0005)
Young's elastic modulus	-12.76 (-113.67, 88.15)	130.69 (-9.84, 271.22)	12.77 (-88.26, 113.80)

Abbreviations: APO, adverse pregnancy outcome; CI, confidence interval; HDP, hypertensive disorders of pregnancy; GDM, gestational diabetes mellitus; PTB, pre-term birth; IMT, intima-media thickness. Multivariable linear regression adjusted for baseline city, mother's age at birth, household income at baseline, primary caregiver's highest educational attainment at any point across the study period, mother smoking during pregnancy, offspring's insurance status at year 22, and offspring's sex.

© 2026 Lam EL et al. *JAMA Network Open*.

eFigure. Histogram of Life's Essential 8 Cardiovascular Health Scores

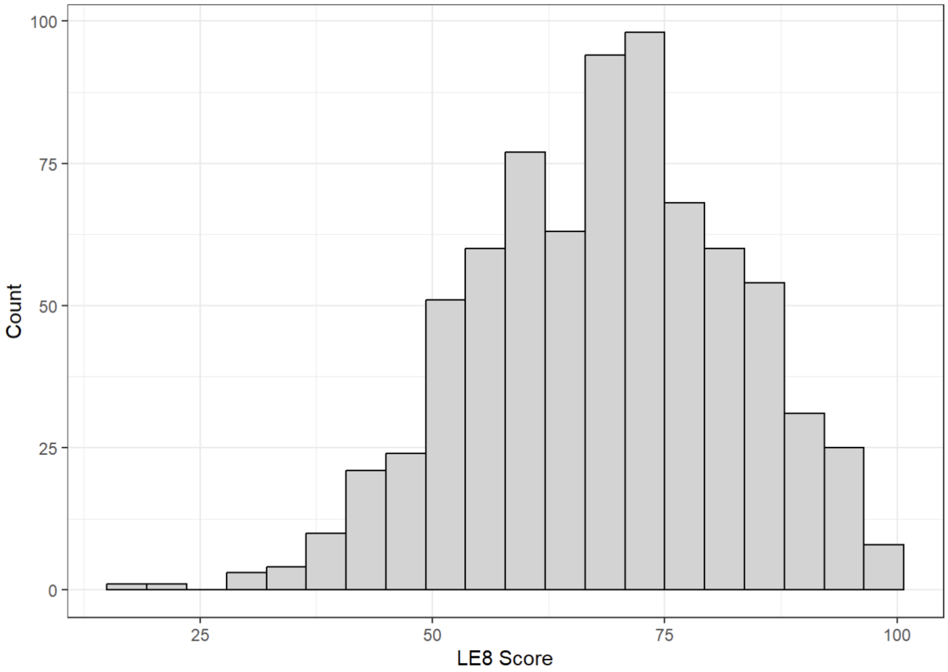

Abbreviations: LE8, Life's Essential 8

© 2026 Lam EL et al. *JAMA Network Open*.
