# Supplementary material for: Adverse Pregnancy Outcomes and Cardiovascular Health Among Offspring in Early Adulthood
Source: JAMA Netw Open. 2026 May 14;9(5):e266783. doi: 10.1001/jamanetworkopen.2026.6783 (PMC13177028; doi:10.1001/jamanetworkopen.2026.6783)
Supplement: Supplement 2. — Data Sharing Statement [file jamanetwopen-e266783-s002.pdf]

Data Sharing Statement

Lam. Adverse Pregnancy Outcomes and Cardiovascular Health Among Offspring in Early Adulthood. *JAMA Netw Open*. Published May 14, 2026.
doi:10.1001/jamanetworkopen.2026.6783

Data
Data available: No

Additional Information
Explanation for why data not available: Data are available through a proposal to the Future of Families and Child Wellbeing Study: https://ffcws.princeton.edu/
